# Distinctive physical insights driven from machine learning modelling of nuclear power plant severe accident scenario propagation

**DOI:** 10.1038/s41598-023-28205-y

**Published:** 2023-01-17

**Authors:** K. Hossny, W. Villanueva, H. D. Wang

**Affiliations:** 1 Nuclear Power Safety (NPS) Division, Department of Physics, School of Engineering Sciences, KTH Royal Institute of Technology, Stockholm, Sweden; 2 Nuclear Futures Institute, School of Computer Science and Electronic Engineering, Bangor University, Bangor, LL57 1UT UK

**Keywords:** Nuclear energy, Power stations, Computational science

## Abstract

The severe accident scenario propagation studies of nuclear power plants (NPPs) have been one of the most critical factors in deploying nuclear power for decades. During an NPP accident, the accident scenario can change during its propagation from the initiating event to a series of accident sub-scenarios. Hence, having time-wise updated information about the current type of accident sub-scenario can help plant operators mitigate the accident propagation and underlying consequences. In this work, we demonstrate the capability of machine learning (Decision Tree) to help researchers and design engineers in finding distinctive physical insights between four different types of accident scenarios based on the pressure vessel's maximum external surface temperature at a particular time. Although the four accidents we included in this study are considered some of the most extensively studied NPPs accident scenarios for decades, our findings shows that decision tree classification could define remarkable distinct differences between them with reliable statistical confidence.

## Introduction

Nuclear reactor safety (NRS) is related to studying possible accident scenarios that can occur in a nuclear power plant (NPP). The purpose of these studies and research is to ensure the safe operation of the NPP. The importance of this field comes from the fact that a single major accident can cause catastrophic damage to the environment, not to mention the risk of losses in personnel. Severe accident scenario propagation has been studied extensively since the dawn of nuclear power generation^1–5^. It has been studied from different points of view, including but not limited to; accidents initiating events and probabilities, instrumentation faults, initiating events propagation, neutronic, and thermal–hydraulic and materials behaviour during different accident scenarios. However, due to the sensitivity and complexity of the subject matter, most of the studies were performed computationally without the use of machine learning (ML). Thanks to the massive advancement in computational power since the mid-2000s, ML applications in various fields have been extensively studied in the past decade. This included broad research areas such as self-driving vehicles, disease detection, explosives detection, stock market behaviour prediction, revolutions and public opinions prediction, etc.^6–11^. In the past three years, much effort has been put into studying ML applications in the NRS field. Most of the ML applications in NRS were oriented toward fault detection and quantification, transients quantification, and improving prediction accuracies by coupling different optimisation techniques with ML models.

H. Wang et al. coupled support vector machines (SVM) and particle swarm optimisation (PSO) to diagnose and analyse different faults in NPPs^12^. They also used kernel principal component analysis (KPCA) to reduce false alarm rates in fault identification by differentiating between the sensors’ malfunctions and anomalies^13^. Liu et al.^14^ tried to use probabilistic support vector regressors (PSVR) to predict the reactor coolant pump (RCP) seal leakage from different internal and external sensors. Zhao et al.^15^ tried detecting failure during normal operation and accident scenarios using a dynamic Bayesian network (DBN). Chae^16^ worked on detecting flow-accelerated corrosion in cooling pipes using SVM, convolutional neural networks (CNN), and long-short term memory (LSTM) models . Nicolau et al.^17^ used a real-time decision tree expert system to identify the root cause of accidents from different sensor readings. Jamil et al.^18^ studied the use of PCA and fisher discriminant analysis (FDA) in identifying control rod withdrawal and external activity insertion faults. Mandal et al.^19^ tried to locate and detect the faulty sensor from a set of sensors’ readings and then classify the fault patterns using singular value decomposition (SVD) and symbolic dynamic filter (SDF). Peng et al.^20^ studied fault detection using deep belief neural networks (DBNN), back propagation neural networks (BPNN), and SVM. Yu et al.^21^ worked on detecting multiple fault detection, isolation, and reconstruction of sensors by coupling PCA with a corrected reconstruction algorithm (CRA) and cyclic PCA (CPCA). Meng et al.^22^ coupled the objective function method (OFM) with an SVM classifier to detect the loose parts in an NPP primary coolant circuit. Ayodeji et al.^23^ used SVR to detect low-level and large leak rates in the steam generator. Li et al.^24^ improved the fault diagnosis accuracies in NPPs by reporting different models' weighted voting and popularity voting methods in ensemble learning models (ELMs). Hadad et al.^25^ worked on classifying design basis accidents using multilayer perceptron (MLP) models. Kim et al.^26^ used a recurrent neural network (RNN) to improve the detection accuracies of multisensor signal measurements. Vadd et al.^27^ used a DBN to classify whether the initiating events were caused due to safety malfunction or a cyber-attack. Mo et al.^28^ studied transient severity quantitively and qualitatively using dynamic neural network aggregation. Mohapatra et al.^29^ studied the possibility of detecting and classifying possible magnetic position sensor faults in the tokamak using different ML techniques such as MLP, K-nearest neighbour (KNN), support vector classifier (SVC), Gaussian naive Bayes (GNB), decision tree classifier (DTC), and random forest classifier (RFC) models.

**Figure 1:**
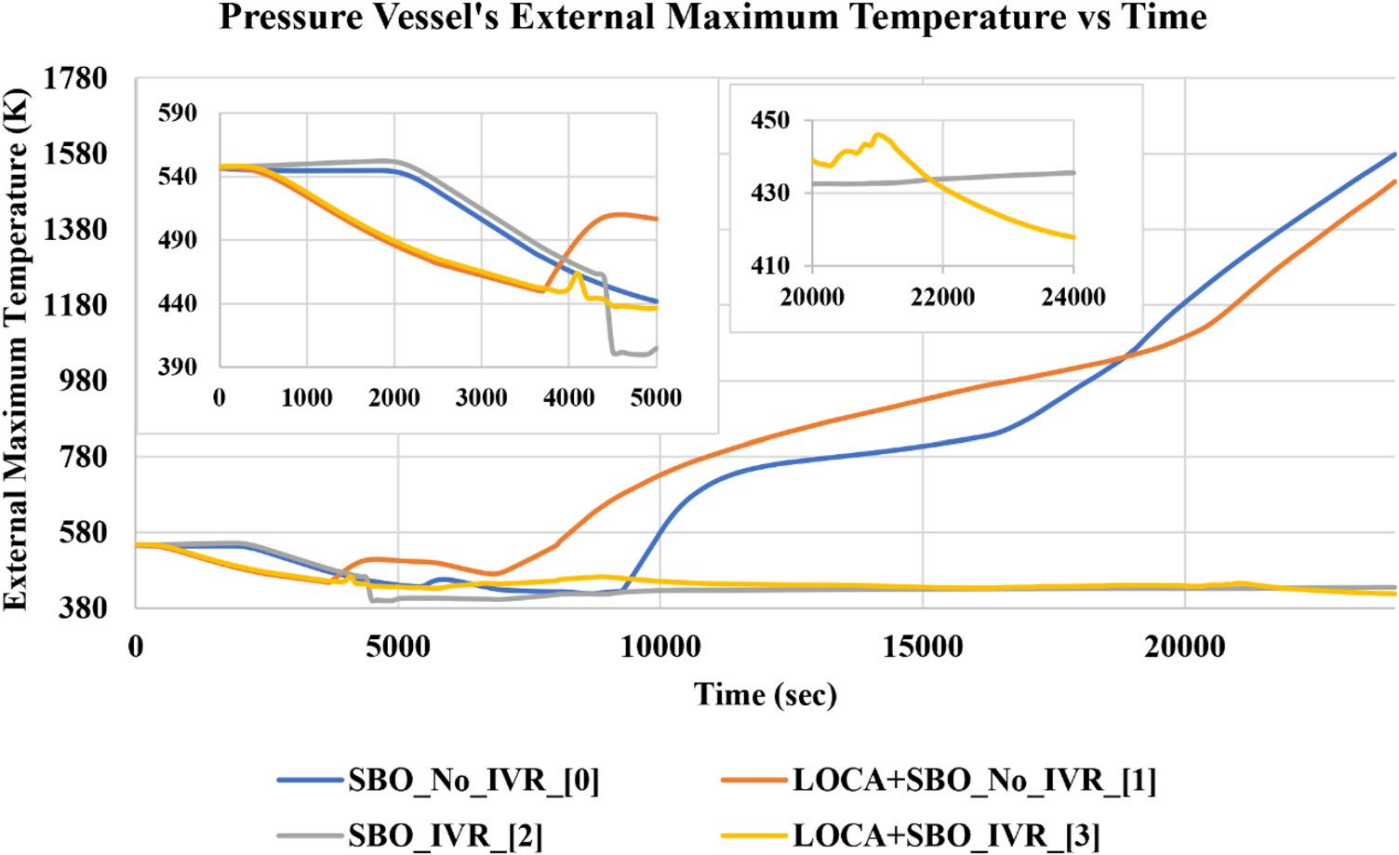
Pressure vessel's external maximum temperature vs time.

The above literature showed the use of ML applications in NRS. It mainly focused on predicting, identifying, or quantifying accident parameters and root causes. In this study, we propose using a DTC algorithm to differentiate between accident scenarios and analyse the data for the studied accident scenarios to gain physical insights into their distinctive differences. We studied how a DTC algorithm would view the data and extract the thresholds that provided its predictive classification decision capabilities. This can point out significant numerical thresholds for each feature that, with further analysis, can point out critical insights that researchers and design engineers have been ignoring or missing. Hence, using the proposed analysis method can provide an extra hand in exploring the data and the distinctive features' thresholds for each accident. We used the time-wise pressure vessel’s maximum external temperature to differentiate between four overlapping accident scenarios in a light water reactor (LWR), which are (1) station blackout (SBO) without in-vessel retention (IVR) by external reactor vessel cooling (ERVC), (2) loss of coolant accident (LOCA) in the presence of SBO without IVR-ERVC, (3) SBO with IVR-ERVC, and (4) LOCA in the presence of SBO with IVR-ERVC. In the cases with IVR-ERVC, external reactor vessel cooling was applied one hour after the accident initiation (time = Zero). The aforementioned accident scenarios will be referred to by their labels of 0, 1, 2, and 3, respectively. It is assumed that external temperature sensors have not been subjected to radiation damage, overheating, or rapid temperature changes. Hence, we chose the maximum external surface temperature as it is more reliable compared to the internal temperature in case of severe accident scenarios. Figure 1 shows the pressure vessel’s time-wise maximum external temperature up to 24,000 s which is the calculated time of failure of the pressure vessel in case of any of the accident scenarios mentioned above. The difference between the temperature behaviour of the accidents in the first 2000s is due to the delay in the automatic depressurization system (ADS) activation in the SBO accidents. The reason behind this delay, in case of SBO accidents, is that the coolant level drop rate must be significant to present the need for activating the ADS to depressurize the reactor’s RPV. It also demonstrates the overlap between the four studied accident scenarios regarding the pressure vessel’s external maximum temperature behaviour. Visually, we can observe the temperature profile differences between accidents 0 and 1 and 2 and 3 at times above 5000 s. However, we cannot follow or calculate the differences between the four accident scenarios at times below 5000 s with proper statistical significance. The same issue is present when trying to find the distinctive differences between accident scenarios labelled 2 and 3 at times higher than 10,000 s. Hence, we used the decision tree (DT) paths of the DTC model we developed to find those distinctive differences. The developed model performance and insights we gathered from the decision paths will be extensively presented and discussed in the “Results and discussion” section. However, model development will be presented and discussed in the “Methods” section. The data provided in Fig. 1. is generated synthetically from MELCOR. MELCOR is an integral code for severe accidents in a NPP, which is employed to simulate a variety of severe accident phenomena^4,30^. In this study, two severe accident scenarios were considered: station blackout (SBO) and a large break ($0.1 {\mathrm{m}}^{2}$ break area at the main steam line) loss-of-coolant accident (LOCA) with station blackout (SBO). In addition, the in-vessel melt retention (IVR) strategy, a postulated mitigation strategy, has been applied to these two accident scenarios. Thus, there are 4 cases to be calculated in MELCOR: SBO and LOCA, with and without an IVR strategy. In each MELCOR simulation, a Nordic BWR with a 3000 MW_th_ nominal power at its original installation is modeled, more details can be found in the references^4^. The thermal–hydraulic response of this reactor is calculated as a function of time for accidents 0 and 1 and 2 and 3, respectively, in MELCOR, including surface temperatures of the vessel, internal, and external pressures. The mesh configuration in each MELCOR simulation generates 28 uneven segments along the vessel's lower plenum. Hence, we obtained the maximum external temperature of the RPV wall at a particular time by comparing the external temperatures in different segments, generating a sub-dataset including two features, 'Time' and 'Temp', for each case. Accordingly, there are 1084 samples in the training dataset.

## Results and discussion

**Figure 2:**
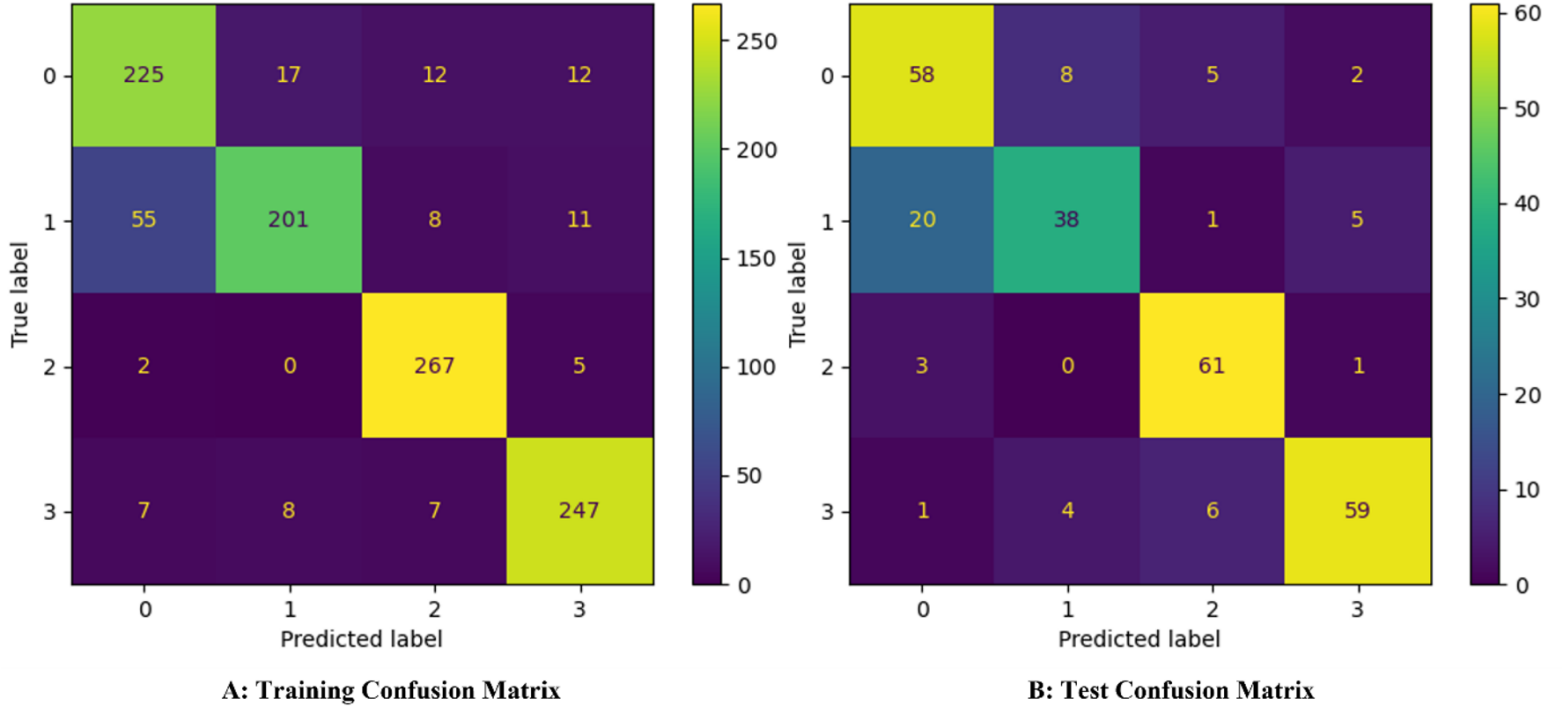
Developed classifier training and test performance confusion matrices.

The developed classifier uses the DT algorithm with 22 minimum samples per leaf. We cross-validated the model performance according to four classification metrics; (1) precision score, (2) accuracy score, (3) f1 score, and (4) recall score. The four metrics were calculated using Eqs. (1)–(4), respectively. $\mathrm{TPC}$, $\mathrm{TNC}$, $\mathrm{FPC}$, and $\mathrm{FNC}$ are true positive class, true negative class, false positive class, and false negative class, respectively. Also, ${\mathrm{w}}_{\mathrm{i}}$, is the samples weight of the $\mathrm{i}$ class in the dataset. We used the Monte Carlo cross-validation method in testing the model’s performance robustness. We performed the cross-validation by training and testing the model over 50 iterations. The initialising seed of the DT algorithm and the samples included in the training and test dataset were randomly changing. Table 1 lists the mean of each training and test metrics and their associated standard deviation and relative error. We calculated the relative error using the formula illustrated in Eq. (5). The motive behind reporting training and test metrics is to ensure the model’s generalisation capability and avoid overfitting. We used the relative error metric as a measure of the standard deviation significance for the training and test metrics which were 1% and 3%, respectively. We selected one of the developed models and analysed its performance metrics, as listed in Table 2. Although it was not one of the top-performing models, after studying its decision-making process, we found that the insights it provides are more general than other top-performing models from the metrics point of view. As mentioned earlier, this is a multi-class problem. Hence, Table 2 lists the performance metrics for each class regarding precision, recall, and f1 scores. The data recorded in Table 2 shows that classes 2 and 3 are the top-performing classes. This is due to the distinctive differences that distinguish each class from the rest in the accident progression, especially within the time range between 10,000 and 24,000 s, as shown in Fig. 1. On the other hand, classes 0 and 1 showed the least performance due to the overlapping behaviour from the beginning to the end of the studied time range. To gain further insights into the model performance, we plotted the confusion matrices of the training and the test data subsets, as shown in Fig. 2. A confusion matrix helps identify which classes have been mislabelled (confused) for another class. The x-axis represents how the model predicted the class, while the y-axis represents the true class of the sample. The numbers allocated in each position represent the number of samples predicted to be correctly or incorrectly labelled. Hence, ideally, for 100% classification metrics, the confusion matrix should only include the number of samples in the diagonal with zeros in the rest. Studying the confusion matrix presented in Fig. 2. we can observe that the most confused classes were class 1, misclassified as class 0. This reflects the nature of overlapping in the behaviour shown in Fig. 1.

**Table 1:** Cross-validated developed models performances.

	Training accuracy	Training precision	Training F1 score	Training recall	Test accuracy	Test precision	Test F1 score	Test recall
Mean	0.86	0.86	0.86	0.86	0.82	0.81	0.81	0.81
Standard deviation	0.01	0.01	0.01	0.01	0.03	0.03	0.03	0.03
Relative error	0.01	0.01	0.01	0.01	0.03	0.03	0.03	0.03

**Table 2:** Selected Model Performance.

	Training accuracy	Training precision	Training F1 score	Training recall	Test accuracy	Test precision	Test F1 score	Test recall
Class 0	0.87	0.78	0.81	0.85	0.79	0.71	0.75	0.79
Class 1		0.89	0.80	0.73		0.76	0.66	0.59
Class 2		0.91	0.94	0.97		0.84	0.89	0.94
Class 3		0.9	0.91	0.92		0.88	0.86	0.84
Mean		0.87	0.87	0.87		0.8	0.79	0.79

1 $$\mathrm{Precision}=\sum_{\mathrm{i}}^{\mathrm{n}}{\mathrm{w}}_{\mathrm{i}}\frac{\mathrm{TPC}}{\mathrm{TPC}+\mathrm{FPC}},$$

2 $$\mathrm{Accuracy}=\frac{\mathrm{TPC}+\mathrm{TNC}}{\mathrm{TPC}+\mathrm{TNC}+\mathrm{FPC}+\mathrm{FNC}},$$

3 $${\mathrm{F}}_{1}\mathrm{ Score}=\sum_{\mathrm{i}}^{\mathrm{n}}{\mathrm{w}}_{\mathrm{i}}\frac{\left.2(\mathrm{Recall}*\mathrm{Precision}\right)}{\mathrm{Recall}+\mathrm{Precision}},$$

4 $$\mathrm{Recall}=\sum_{\mathrm{i}}^{\mathrm{n}}{\mathrm{w}}_{\mathrm{i}}\frac{\mathrm{TPC}}{\mathrm{TPC}+\mathrm{FNC}},$$

5 $$\mathrm{Relative\ Error}= \frac{\mathrm{Standard\ Deviation}}{\mathrm{Mean}},$$

**Figure 3:**
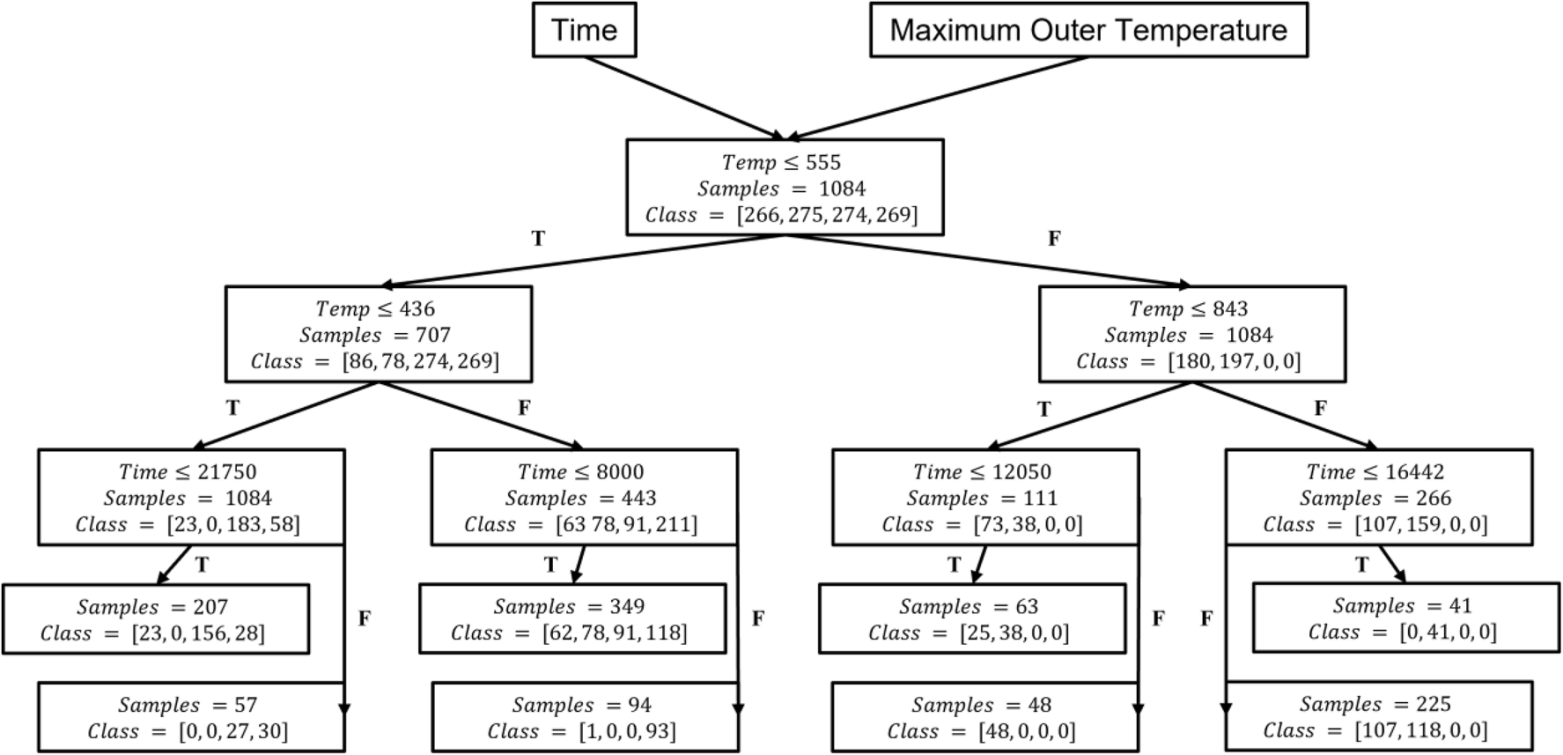
Four layers of the selected model DT.

We studied the first four layers of the DT of the selected model to extract the Thresholds upon which the algorithm chose to split and classify the data, as shown in Fig. 3. The term ’Samples’ reflects the number of studied samples, and the set called ‘Classes’ represents the number of samples in each class of 0, 1, 2, and 3, respectively. This study aims to show the potential for using ML predictive capabilities in exploratory data analysis. Hence, the numerical values of the thresholds we extracted from the developed model were specified by the DT algorithm, which is discussed in detail in the “Methods” section. These thresholds represent the critical indicators upon which we can classify the different accident scenarios. Although the bottom of the decision tree will have more pure nodes and leaves in which several leaves will represent each class. However, we chose to study the thresholds that form more significant splits. These kinds of splits are present at the top of the decision tree. The input was the time and the pressure vessel maximum external temperature represented in the figure by the terms ‘Time’ and ‘Temp’, respectively. At each node, the model checks whether the sample’s time or temperature satisfies the condition at the node or not (true (T) or false (F)). Based on that, the sample proceeds to the next Threshold. These conditions were determined based on the DT algorithm during the model development. We extracted these thresholds to study how the DT algorithm classifies between the different classes (accidents). We reported all (training and test data sets) the predicted class outcomes from the developed model. We studied which path they followed to calculate the prediction accuracy for each path, as listed in Table 3 where column ‘Label’ denotes the label of the path, which represents a chain of thresholds that the sample must satisfy to fall within the path. From Table 3, we observed that the most reliable paths were the ones labelled P1, P2, P4, P6, and P7, as these paths’ accuracies were higher than 90% in both training and test. Hence, we calculated their weighted accuracies weighted on the number of samples in both training and test datasets, as listed in Table 4.

**Table 3:** DT paths training and test accuracies.

Label	Threshold	Training accuracy	Test accuracy
P1	$\mathrm{Temp}\le 436$	0.95	0.96
	$$\mathrm{Time}\le 21750$$		
P2	$\mathrm{Temp}\le 436$	0.98	0.94
	$$\mathrm{Time}>21750$$		
P3	$436<\mathrm{Temp}\le 555$	0.8	0.68
	$$\mathrm{Time}\le 8000$$		
P4	$436<\mathrm{Temp}\le 555$	0.99	1
	$$\mathrm{Time}>8000$$		
P5	$555<\mathrm{Temp}\le 843$	0.67	0.5
	$$\mathrm{Time}\le 12050$$		
P6	$555<\mathrm{Temp}\le 843$	1	1
	$$\mathrm{Time}>12050$$		
P7	$\mathrm{Temp}>843$	1	1
	$$\mathrm{Time}\le 16442$$		
P8	$\mathrm{Temp}>843$	0.82	0.7
	$$\mathrm{Time}>16442$$

**Table 4:** Weighted paths accuracies.

Label	P1	P2	P3	P4	P5	P6	P7	P8
Weighted path accuracy	0.95	0.97	0.78	0.99	0.64	1	1	0.8

**Figure 4:**
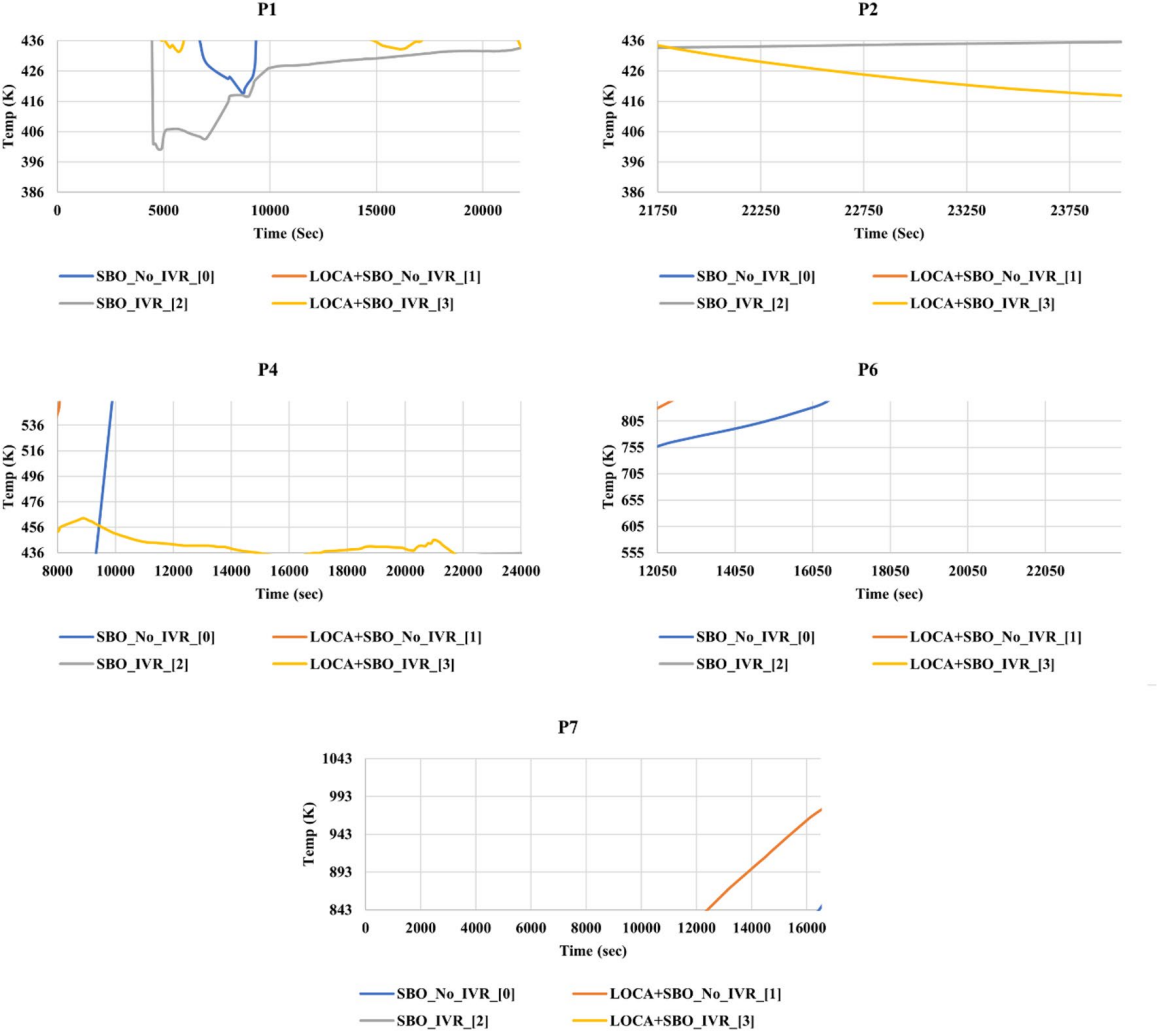
Applying thresholds of each path to the time-wise accident progression behaviour.

Weighted path accuracies listed in Table 4 represent how many samples were predicted correctly by applying the series of thresholds of each path. We used this metric to measure the reliability of the sequential thresholds forming each path. Hence, we chose to study the paths that scored weighted accuracies higher than 90%. Finally, we applied the chosen paths’ thresholds on the four studied accidents' time-wise propagation to study the insights they provided, as shown in Fig. 4. The five subfigures are labelled with the path threshold applied to the original behaviour shown in Fig. 1. We observed that P1 thresholds did not provide significant distinctions for the studied accidents. However, it provided a negative insight that in the range of time and RPV’s external surface temperature specified by P1, we observed that the LOCA in the presence of an SBO accident without the activation of IVR could not occur under these thresholds. Hence, we can use this information to confirm if the IVR system has been activated or not in this accident scenario. In the P2 subfigure, we can observe that the common feature in the two accidents that satisfy the associated thresholds with P2 is the presence of IVR. However, these insights are late in time when damage has already been done to the reactor core. Although, the trained model showed poor performance in classifying accident scenarios labelled 0, and 1 as mentioned earlier. Nevertheless, our proposed method of analysis showed that it can point out key thresholds in exploring the accident scenario data to provide insights about distinctive differences between the progression of the two classes as illustrated in P1 and P2. However, this can provide helpful information in the post-accident scenario analysis to study the reasons behind a specific accident progression. Such as, the ‘Time’ threshold is 21,750 s, corresponding to the RPV ablation phenomenon that happens around 6.17 h from the accident’s (without IVR activation) initiation time^1^. The thresholds in the P4 path leading to subplot P4 denote that the temperature is still low relatively after 8000 s, corresponding mainly to the LOCA in the presence of SBO with IVR system activation. This is yet another indication of successful activation of the IVR system as the core support plate in accident scenarios without IVR (i.e., accidents 0 and 1) will fail at time 7913 s and 6183 s, respectively^4^, indicating the start of corium relocation to RPV lower head. Consequently, the temperature of the RPV wall will increase due to the heating from this hot corium. With the IVR strategy’s successful activation (i.e., accidents 3 and 4), the temperatures of RPV walls are lower, in particular for LOCA in the presence of SBO with IVR system, in which the pipe breach and the early activation of ADS accelerate coolant vaporization leading to the decrease of heat inside the RPV. However, we observed the presence of another accident that satisfied the exact thresholds of P4. This indicates that an expert opinion must validate the insights and improve the thresholds extracted from the DT algorithm. Similarly, subplots P6 and P7 show that the dominant accident scenarios are SBO and LOCA in the presence of SBO, both without IVR system activation, respectively. Although Fig. 3 points out that only class 0 and class 1 can occur while applying the P6 and P7 thresholds, respectively. However, subplots P6 and P7 in Fig. 4 show the presence of class 1 and 0, respectively. This observation is because sample points taken in the training and test during the model development and threshold extraction are discretized points. However, interpolated points appear when applying the extracted thresholds on a spline curve of the accident progression. The insights we gathered from the developed DT model can be the cornerstone for causality analysis. This can help in having a more profound understanding of the physical causes of accident scenario propagation. Such understanding can help develop better operation and safety systems, materials, and accident scenario mitigation protocols.

## Conclusions

In this work, we presented the need for the inclusion of ML in accident scenario classification as it is urgent to know the accident deviation from the initiating event to be able to follow the proper protocols in accident scenario mitigation to avoid severe consequences such as what happened during the Fukushima-Daiichi accident in 2011. This work also showed the potential of using ML algorithms not only in the development of predictive models capable of predicting the accident type, but also as an exploratory analysis tool that can provide distinctive insights about how we can differentiate between the different accident scenarios based on the time-wise propagation of their physical phenomena (in our case, the maximum external temperature of the reactor pressure vessel). However, the insights provided by the ML algorithm must be cross-validated by researchers and design engineers for a sensitive and complex subject such as NRS. Hence, these insights can help the subject matter expert in having a more profound understanding of the differences between accident scenarios propagation. Although the case study we presented in this work includes four accident scenarios, it is a proof of concept to show the ML capabilities in exploratory analysis. Future work will include the application of the same approach we are proposing in this work to a more significant number of accident scenarios when it is impractical to visually inspect the different behaviours of hundreds of accident scenarios and sub-scenarios. Furthermore, inclusion of the structural analysis of the lower head of the pressure vessel will provide additional insights to the structural integrity of the pressure vessel at any particular time.

## Methods

### Decision tree classification theoretical background

**Figure 5:**
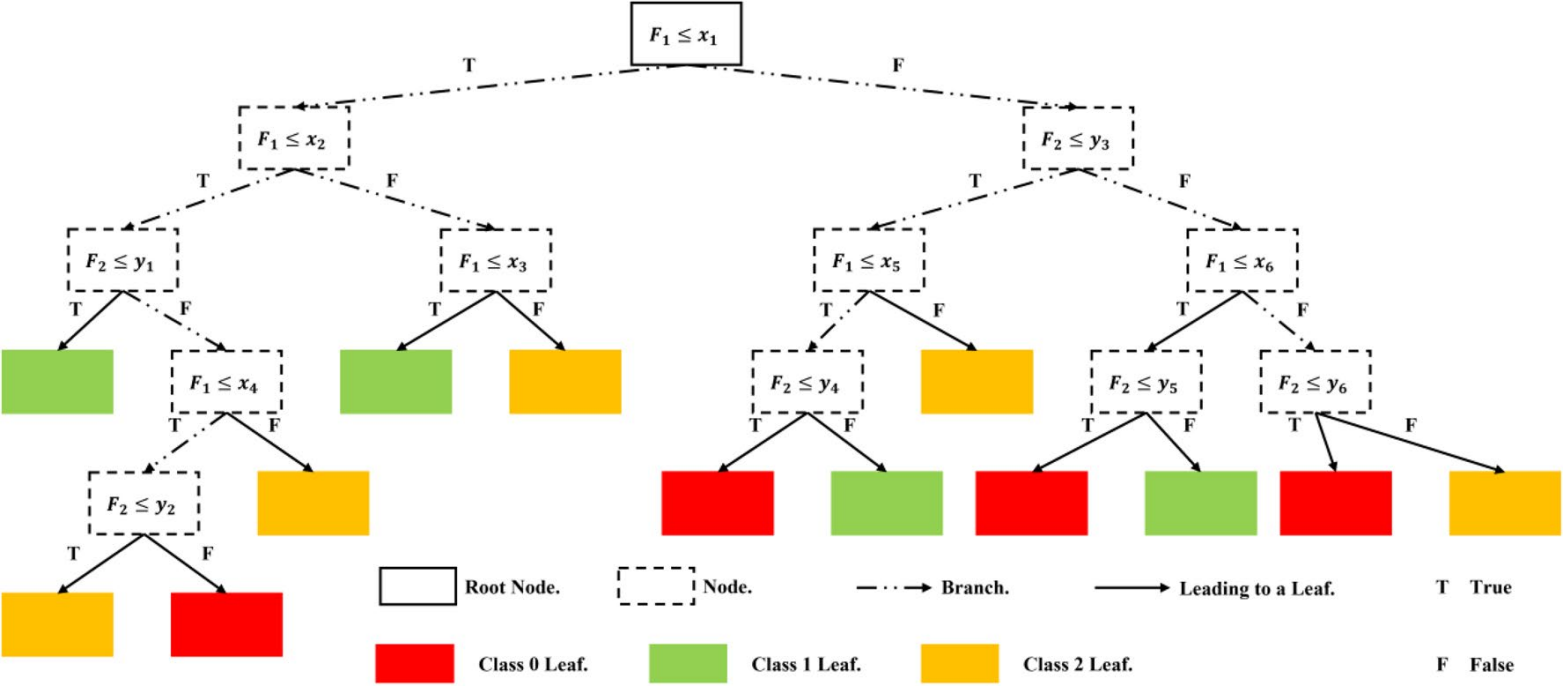
DTC example schematic.

Decision tree classification (DTC) is a stochastic-initiated ML algorithm^31^. It consists of a root node, branches, nodes, and leaves, sequentially, as illustrated in Fig. 5. The example illustrated in Fig. 5 shows a DTC that takes two inputs (${\mathrm{F}}_{1}$, and ${\mathrm{F}}_{2}$) and tries to differentiate between three output classes (red, green, and yellow). The algorithm depends on defining a series of interconnected thresholds for the input features. The purpose of defining the thresholds is to reduce the impurity of successive nodes until reaching almost pure leaves. The impurity of each node is quantified using the ‘Gini Impurity Index’ or ‘Entropy’ which can be calculated using Eqs. (6), and (7), respectively. ${\mathrm{G}}_{\mathrm{I}}$, and $\mathrm{E}$ are the ‘Gini Impurity Index’, and ‘Entropy’, respectively, while ${\mathrm{p}}_{\mathrm{i}}$, and $\mathrm{k}$ are the probability of each class presence in the data set, and the number of the classes, respectively. Quantifying the node impurity reduction is performed by calculating the ‘Information Gain’ between parent and daughter nodes or leaves using Eq. (8). $\mathrm{IG}$ is the ‘Information Gain’, ${\mathrm{I}}_{\mathrm{P}}$, ${\mathrm{I}}_{\mathrm{left}}$, ${\mathrm{I}}_{\mathrm{right}}$ are the Impurity Indices of the parent, left daughter, and right daughter nodes, respectively, while $\mathrm{N}$, ${\mathrm{N}}_{\mathrm{left}}$, ${\mathrm{N}}_{\mathrm{right}}$ are the number of samples in the complete dataset, left daughter node, and right daughter node, respectively.

6 $${\mathrm{G}}_{\mathrm{I}}=1-{\sum }_{\mathrm{i}=1}^{\mathrm{k}}{\mathrm{p}}_{\mathrm{i}}^{2},$$

7 $$\mathrm{Entropy }\left(\mathrm{E}\right)=-{\sum }_{\mathrm{i}=1}^{\mathrm{k}}{\mathrm{p}}_{\mathrm{i}}{\mathrm{log}}_{2}\left({\mathrm{p}}_{\mathrm{i}}\right),$$

8 $$\mathrm{IG}={\mathrm{I}}_{\mathrm{P}}-\left[\left(\frac{{\mathrm{N}}_{\mathrm{left}}}{\mathrm{N}}*{\mathrm{I}}_{\mathrm{left}}\right)+\left(\frac{{\mathrm{N}}_{\mathrm{right}}}{\mathrm{N}}*{\mathrm{I}}_{\mathrm{right}}\right)\right],$$

### Model development

**Figure 6:**
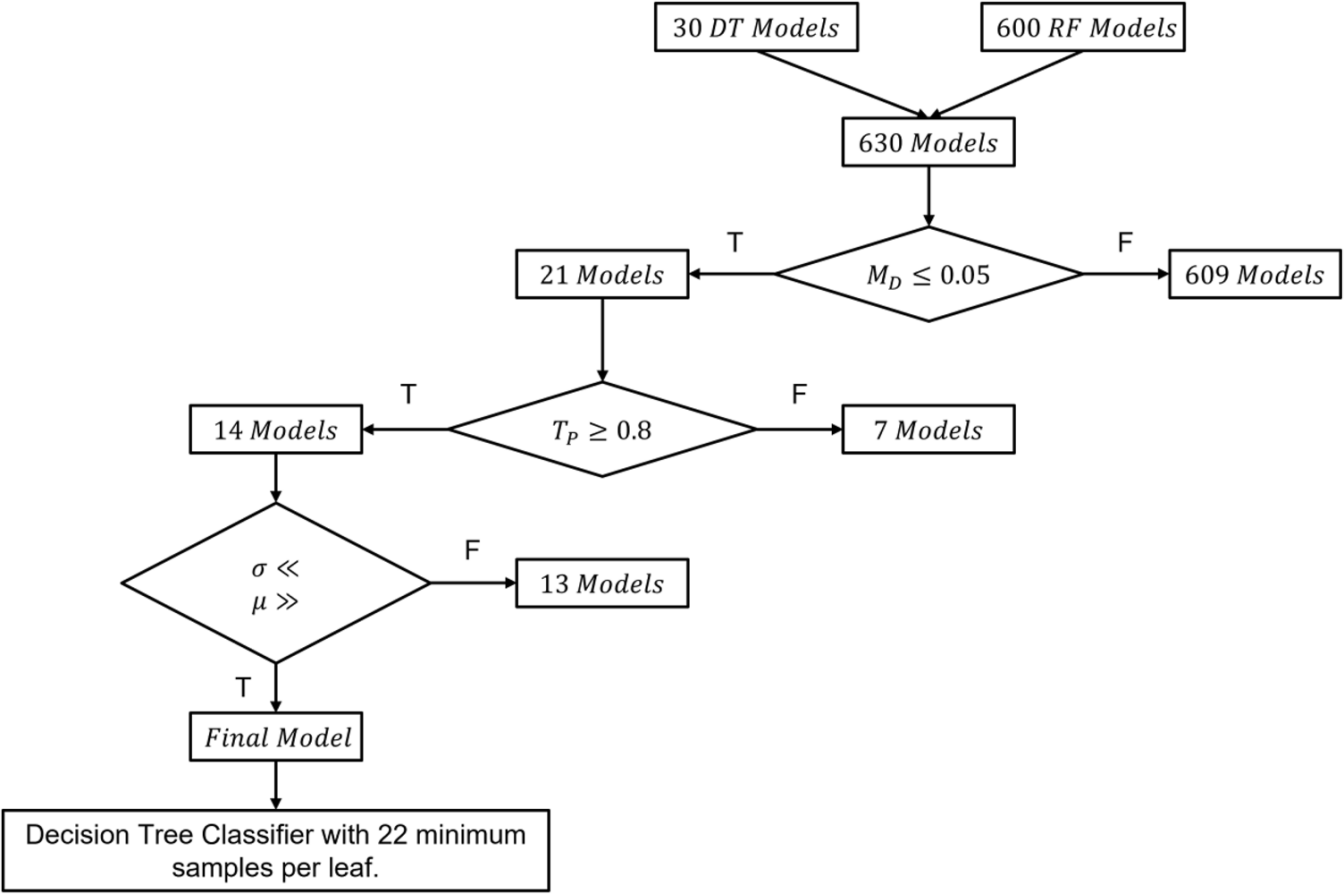
Hyperparameters selection method.

To select the optimal model for our classification problem. We tried several classification algorithms with their defaults parameters and reported their respective training and test classification metrics, as listed in supplementary Table 1. From the models’ performances summarized in supplementary Table 1, we chose decision tree, and random forest classification (RFC) techniques to go through the process of hyperparameters tuning. The reason behind choosing these two techniques is that they showed the least difference between training and test performance metrics as listed in Table 5. We used the difference between training and test performance metrics as an indication of a lower likelihood of overfitting. To select the optimal model, we started the hyperparameters selection process. We found the most influential hyperparameters in the DTC and RFC techniques to be the minimum number of samples per leaf, and minimum samples per leaf and the number of estimators, respectively. Hence, we tried to vary the number of minimum samples per leaf from one to 30 for both algorithms. We also varied the number of estimators in the RFC algorithm from 1 to 96 with an increment of five. These variations yielded 30 DTC models and 600 RFC models. We reported training and test metrics for each model. We performed the optimal model selection by following the steps illustrated in Fig. 6. The selection process consisted of a series of sequential steps. First, eliminating the models that have a higher likelihood of overfitting by calculating the difference between the model’s training and test precisions. If it is higher than 5%, then the model is eliminated. This process successfully dropped the number of models from 630 to 21. Second, to ensure the selected models are not underfitting, we dropped the modes with test performance precision metrics of less than 80%. Finally, to ensure the selected model is highly robust, we cross-validated the remaining 14 models over 30 folds by randomly splitting the training and test data. In each fold, the training, and test datasets represented 80% and 20% of the whole dataset, respectively. We then selected the model that provided the highest mean test performance metrics with the least associated standard deviation.

**Table 5:** Different ML models training and test performance metrics.

Model	Training metrics				Test metrics			
	Precision	Accuracy	F1-Score	Recall	Precision	Accuracy	F1-Score	Recall
Decision Tree	1	1	1	1	0.91	0.91	0.91	0.91
Random Forest	0.79	0.8	0.79	0.8	0.74	0.75	0.74	0.75

## Supplementary Information

Supplementary Table 1.

## Data Availability

The data that support the findings of this study are available from [KTH Royal Institute of Technology] but restrictions apply to the availability of these data, which were used under license for the current study, and so are not publicly available. Data are however available from the authors upon reasonable request and with permission of [KTH Royal Institute of Technology]. Please contact the corresponding author for the raw data.
