# Supplementary material for: Distinctive physical insights driven from machine learning modelling of nuclear power plant severe accident scenario propagation
Source: Sci Rep. 2023 Jan 17;13:930. doi: 10.1038/s41598-023-28205-y (PMC9845314; doi:10.1038/s41598-023-28205-y)
Supplement: Supplementary file 1 — Supplementary Table 1. [file 41598_2023_28205_MOESM1_ESM.pdf]

Distinctive Physical Insights Driven from Machine Learning Modelling of Nuclear Power Plant Severe Accident Scenario Propagation

K. Hossny[*1], W. Villanueva[1,2], and H. D. Wang[1]

[1] Nuclear Power Safety (NPS) Division, Department of Physics, School of Engineering Sciences, KTH Royal Institute of Technology, Stockholm, Sweden.
[2] Nuclear Futures Institute, School of Computer Science and Electronic Engineering, Bangor University, LL57 1UT Bangor, United Kingdom.

[*] Corresponding Author E-Mail: kmmhma@kth.se

Supplementary Table 1. Different ML Models Training and Test Performance Metrics

**Model**	**Training Metrics**				**Test Metrics**			
	**Precision**	**Accuracy**	**F1-Score**	**Recall**	**Precision**	**Accuracy**	**F1-Score**	**Recall**
**Decision Tree**	1	1	1	1	0.91	0.91	0.91	0.91
**Random Forest**	0.79	0.8	0.79	0.8	0.74	0.75	0.74	0.75
**K Neighbours**	0.79	0.78	0.77	0.78	0.61	0.60	0.59	0.60
**Quadratic Discriminant Analysis**	0.62	0.59	0.58	0.59	0.61	0.57	0.56	0.57
**Adaptive Boosting**	0.51	0.53	0.45	0.53	0.39	0.47	0.38	0.47
**Gaussian Naive Bayes**	0.50	0.49	0.46	0.49	0.50	0.47	0.45	0.47
**Gaussian Process**	1	1	1	1	0.47	0.29	0.27	0.29
**Multilayer Perceptron**	0.30	0.37	0.25	0.37	0.25	0.42	0.30	0.42
